# Natural products and synthetic analogs as selective orphan nuclear receptor 4A (NR4A) modulators

**DOI:** 10.14670/HH-18-689

**Published:** 2023-12-13

**Authors:** Stephen Safe

**Affiliations:** Department of Veterinary Physiology and Pharmacology, Texas A&M University, College Station, TX, USA

**Keywords:** NR4A1, NR4A2, NR4A3, Ligands, Binding, Functions, Selective

## Abstract

Although endogenous ligands for the orphan nuclear receptor 4A1 (NR4A1, Nur77), NR4A2 (Nurr1), and NR4A3 (Nor-1) have not been identified, several natural products and synthetic analogs bind NR4A members. These studies are becoming increasingly important since members of the NR4A subfamily of 3 receptors are potential drug targets for treating cancer and non-cancer endpoints and particularly those conditions associated with inflammatory diseases. Ligands that bind NR4A1, NR4A2, and NR4A3 including Cytosporone B, celastrol, bis-indole derived (CDIM) compounds, tryptophan/indolic, metabolites, prostaglandins, resveratrol, piperlongumine, fatty acids, flavonoids, alkaloids, peptides, and drug families including statins and antimalarial drugs. The structural diversity of NR4A ligands and their overlapping and unique effects on NR4A1, NR4A2, and NR4A3 suggest that NR4A ligands are selective NR4A modulators (SNR4AMs) that exhibit tissue-, structure-, and response-specific activities. The SNR4AM activities of NR4A ligands are exemplified among the Cytosporone B analogs where n-pentyl-2-[3,5-dihydroxy-2-(nonanoyl)]phenyl acetate (PDNPA) binds NR4A1, NR4A2 and NR4A3 but activates only NR4A1 and exhibits significant functional differences with other Cytosporone B analogs. The number of potential clinical applications of agents targeting NR4A is increasing and this should spur future development of SNR4AMs as therapeutics that act through NR4A1, NR4A2 and NR4A3.

## Introduction

The human nuclear receptor (NR) family contains 48 individual receptors that mediate signals induced by endogenous and exogenous ligands that are critically important for maintaining cellular homeostasis and pathophysiology (Bookout et al., 2006; Sonoda et al., 2008). NRs have been divided into categories based, in part, on their ligands and include the endocrine receptors which bind hormones/vitamins, adopted orphan receptors which bind lipids and steroid-derived small molecules and orphan receptors for which endogenous ligands have not yet been identified (Bookout et al., 2006; Sonoda et al., 2008). These receptors regulate both diverse and overlapping pathways and their commonality is not based on function but on structure and activation mechanisms. Nuclear receptors express an N-terminal activation function-1 (AF1), a DNA-binding domain (DBD), a hinge region and a C-terminal ligand binding domain (LBD) which also contains AF2. In the absence of ligand, the receptor or receptor complex may or may not be associated with its cognate DNA response element on responsive gene promoters; however, upon ligand binding, the DNA bound receptors modulate gene expression. The effects of ligands for NR4A and their mechanisms of action will subsequently be reviewed in detail. The major exceptions to these common features of orphan NRs are the DAX1 (NROB1) and SHP (NROB2) receptors that do not contain a DBD and act primarily through protein-protein interactions (Seol et al., 1996; Lalli and Sassone-Corsi, 2003; Ehrlund and Trenter, 2012).

### Functions of NR4A

The nerve growth factor β (NGFβ) or NR4A subfamily denotes a group of 3 receptors, namely NR4A1 (Nur77, TR3), NR4A2 (Nurr1) and NR4A3 (Nor1). These receptors were initially discovered as immediate early response genes that were induced by nerve growth factor in PC12 and other neuronal cells (Milbrandt, 1988; Law et al., 1992; Ohkura et al., 1994). Results of gene deletion experiments show that NR4A1^−/−^ mice are viable with some impaired regulation of T cells but no distinct phenotype (Woronicz et al., 1994; Lee et al., 1995; Cheng et al., 1997) whereas NR4A2^−/−^ mice exhibit neurological dysfunction associated with the dopaminergic system and embryos die within one week (Zetterström et al., 1997; Saucedo-Cardenas et al., 1998). Two laboratories generated NR4A3^−/−^ mice which exhibit divergent phenotypes; one set of NR4A3^−/−^ mice exhibits embryo lethality associated with failure to complete gastrulation whereas the other set of embryos is viable and exhibits inner ear defects (Ponnio et al., 2002; De Young et al., 2003). There have been extensive studies on the dual NR4A1^−/−^ : NR4A3^−/−^ knockout mice in which the combined loss of both genes results in the rapid development of acute myeloid leukemia in the offspring (Mullican et al., 2007; Ramirez- Herrick et al., 2011).

**Fig. 1.**
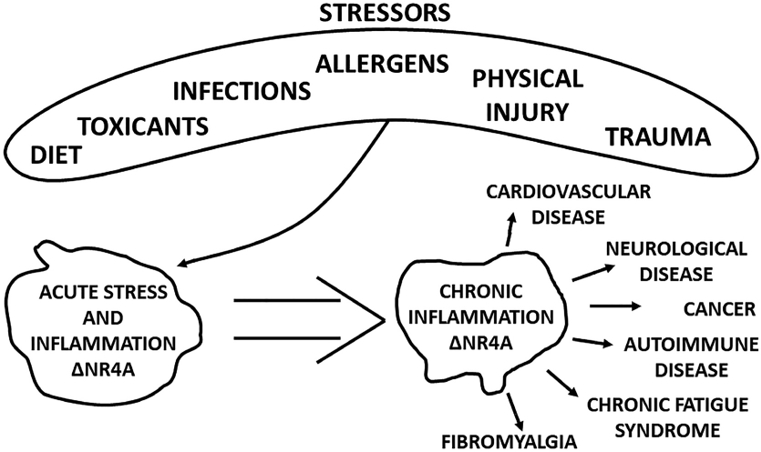
Stressors, inflammation and NR4A members. Multiple stressors induce NR4A to maintain cellular homeostasis (Maxwell and Muscat, 2006; Pearen and Muscat., 2010) and the subsequent development of acute and chronic inflammation and associated diseases are often accompanied by increased levels of NR4A genes/gene products (Bonta et al., 2006; Pei et al., 2006; Hawk et al., 2012; McNulty et al., 2012; Close et al., 2013; Beard et al., 2015; Niu et al., 2015; Palumbo-Zerr et al., 2015; Liebmann et al., 2018; Wu and Chen, 2018; Chen et al., 2020; Lith and Vries, 2020; Xiong et al., 2020; Lith et al., 2021; Safe and Karki, 2021).

The endogenous functions of NR4A1, NR4A2, and NR4A3 and their role in diseases have been extensively investigated and ongoing research continues to add to their diverse functional roles. NR4A members are induced or modified by a variety of stressors as illustrated in Figure 1 and this response plays a role in coping with these stressors and maintaining cellular homeostasis (Maxwell and Muscat, 2006; Pearen and Muscat, 2010). NR4A1, NR4A2 and NR4A3 play significant roles in metabolism, the immune system, cardiovascular and neurological functions such as learning and memory and wound healing (Maxwell and Muscat, 2006; Pearen and Muscat, 2010; Lith and Vries, 2020; Chen et al., 2020; Lith et al., 2021). A result of long-term stressor-induced responses can lead to acute and ultimately chronic inflammatory diseases (Fig. 1) which include cardiovascular, neurological, and autoimmune diseases, rheumatoid arthritis, cancer, lupus, fibromyalgia, and chronic fatigue syndrome. Not surprisingly, NR4A members are overexpressed in many solid tumors and stress/inflammation-induced diseases (Bonta et al., 2006; Pei et al., 2006; Hawk et al., 2012; McNulty et al., 2012; Close et al., 2013; Beard et al., 2015; Niu et al., 2015; Palumbo-Zerr et al., 2015; Liebmann et al., 2018; Wu and Chen, 2018; Chen et al., 2020; Lith and Vries, 2020; Xiong et al., 2020; Lith et al., 2021; Safe and Karki, 2021). For example, transforming growth factor β-induced fibrosis in multiple tissues is linked to crosstalk with NR4A1 which results in inhibition of TGFβ signaling that is further enhanced after treatment with the NR4A1 ligand Cytosporone B (CsnB) (Palumbo-Zerr et al., 2015). In many solid tumors NR4A1 is overexpressed including lung, breast, ovarian, and colon cancer, and NR4A1 is also a negative prognostic factor for patient survival (Smith et al., 2011; Lee et al., 2012; Wang et al., 2014a; Zhou et al., 2014; Delgado et al., 2016). Moreover, functional studies show that both NR4A1 and NR4A2 are pro-oncogenic in most solid tumors and can be targeted by synthetic ligands that act as inverse NR4A1/2 agonists and inhibit NR4A1- and NR4A2-regulated pro-oncogenic genes and their associated pathways (Safe and Karki, 2021); corresponding results for NR4A3 are limited. Initial studies on the structure of the ligand binding domain of NR4A used NR4A2 as a model and the results of crystal structure studies indicated that bulky amino acid side chains protruded into the ligand binding domain and would inhibit ligand binding (Wang et al., 2003; Wansa et al., 2003; Flaig et al., 2005). These and other observations suggested that NR4A functions were ligand-independent. This tempered studies on discovery of endogenous and synthetic NR4A ligands; however, there are now extensive reports on the identity and functions of natural products and synthetic analogs that bind NR4A1, NR4A2 and NR4A3 (to a lesser extent) and their possible chemotherapeutic applications. It should also be noted that ligands for many nuclear receptors exhibit tissue/cell- and response-specific activities as agonists, inverse agonists and antagonists and are classified as selective receptor modulators (Jordan, 2003; Burris et al., 2013). There is also evidence for NR4A ligands as selective NR4A modulators (SNR4AMs) and this will also be discussed.

## NR4A ligands as selective NR4A modulators (SNR4AMs)

**Fig. 2.**
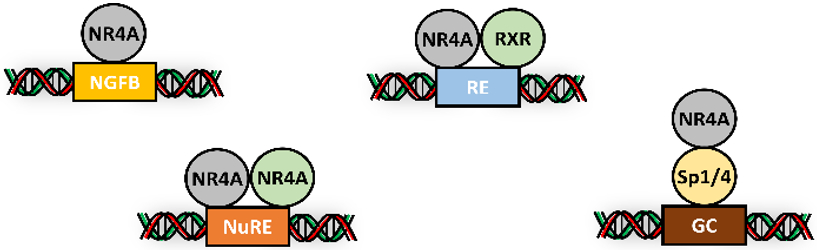
NR4A as a transcription factor. NR4A members bind cognate response elements as monomers, dimers, heterodimers (with RXR) and interact with DNA-bound Sp proteins as nuclear cofactors.

Like other NRs, NR4A1, NR4A2 and NR4A3 exhibit a modular structure in which there is 97% homology in their DBDs and 60-65% homology in their LBDs and only 20-30% homology in their N-terminal AF1 domain (Maruyama et al., 1998). This suggests that many of the differences in function may be due, in part, to AF1 domain and its interactions with other nuclear co-factors and the transcriptional machinery. NR4A interacts with cis-promoter elements as monomers and homodimers binding to an NBRE (-AAAGGTCA) and a Nur response element (NuRE) (TGATATTTX6A AAGTCA) respectively (Wilson et al., 1991, 1992; Paulsen et al., 1995; Perlmann and Jassson, 1995; Zetterström et al., 1996; Philips et al., 1997) (Fig. 2). In addition, both NR4A1 and NR4A2 form heterodimers with the retinoid X receptor (RXR) and NR4A interacts with p65 of NFKβ to trans-repress NFKβ-dependent activation (McEvoy et al., 2002; Calvayrac et al., 2015; Popichak et al., 2018; Zhang et al., 2019; Lilley et al., 2022). Another similar mechanism involves interaction of NR4A1 with either Sp1 or Sp4 and the NR4A1/Sp1 complex activates expression of some genes through their GC-rich promoters (Lee et al., 2010; Li et al., 2012; Hedrick et al., 2017; Lacey et al., 2017; Karki et al., 2020b; Shrestha et al., 2021a) (Fig. 2). This pathway of gene regulation where NR4A1 acts as a cofactor is also observed for many other nuclear receptors (Safe and Kim, 2008).

### The concept of selective receptor modulators

Drugs targeting NRs and other receptors are among the most abundant and widely used pharmaceutical agents, and this is due, in part, to their ability to selectively activate agonist, inverse agonist, or antagonist activities. For example, the anticancer drug tamoxifen binds the estrogen receptor (ER) and exhibits ER antagonist activity against human breast cancer but is an ER agonist in the uterus (Zhou et al., 2014). In contrast, the antiestrogen fulvestrant also binds ER and acts as an inverse ER agonist and also induces proteasome dependent degradation of ER in breast cancer (Wang et al., 2004). In addition, fulvestrant induces some ER-regulated genes including p21 and quinone reductase and this may involve both direct and indirect mechanisms of action (Montano and Katzenellenbogen,1997; Varshochi et al., 2005). These results showing that ER ligands can exhibit multiple ER-dependent activities is due to several factors including ligand structure- dependent interaction with ER resulting in confirmational changes, tissue-specific expression of cofactors and chromatin interactions/modifications (Jordan, 2003; Burris et al., 2013). These factors can modify the effects of ligand-receptor interactions in terms of their agonist, inverse agonist and antagonist activities and evidence for selective NR4A modulator activity of NR4A ligands will be examined in this review.

Several studies on chemical-induced activation of primarily NR4A1 demonstrate that several apoptosis inducing agents (e.g., phorbol esters) and retinoids induce nuclear export of the receptor which forms a proapoptotic NR4A1-bcl2 complex (Li et al., 1998, 2000; Pfahl and Piedrafita, 2003; Holmes et al., 2004; Lin et al., 2004). Other agents also induce nuclear export of NR4A1 which subsequently interacts with multiple mitochondrial and extra-mitochondrial factors which usually result in the induction of cell death. Initial studies showed that these effects were kinase-dependent but were not activated by NR4A1 ligands (Li et al., 1998; Li et al., 2000; Pfahl and Piedrafita, 2003; Holmes et al., 2004; Lin et al., 2004) however, subsequent reports show that some NR4A1 ligands can also induce kinase-dependent activation of nuclear export of NR4A1, and this has recently been reviewed (Safe and karki, 2021). This review will primarily focus on ligands that directly bind and activate nuclear NR4A, however for some ligands this is accompanied by nuclear export pathways.

### 6-Mercaptopurine (Fig. 3)

**Fig. 3.**
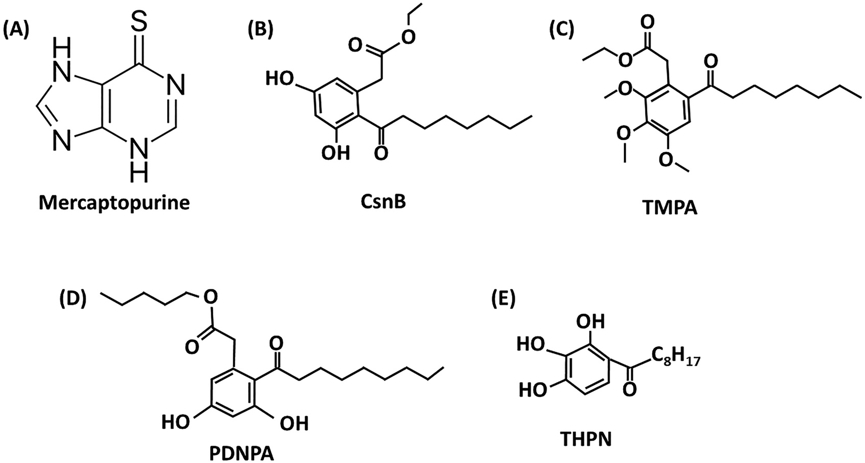
Structures of 6-MP **(A)**, CsnB **(B)** and related analogs TMPA **(C)**, PDNPA **(D)** and THPN **(E)**.

6-Mercaptopurine (6-MP) (Fig. 3) an inhibitor of purine biosynthesis and has been used to treat acute childhood leukemia and chronic myelocytic leukemia. Initial studies showed that 6-MP activated both NR4A2 and NR4A3 and this response was dependent on the N-terminal AF1 domain of both receptors (Wansa and Muscat, 2003; Smith et al., 2011). Subsequent studies showed that interaction with the TRAP220 coactivator enhanced 6- MP-dependent activation of NR4A3. NR4A1 also interacted with TRAP220 however, the effects of 6-MP were not reported (Ordentlich et al., 2003; Wansa and Muscat, 2005). Thus 6- MP activated NR4A2, NR4A3, and possibly NR4A1 via the N-terminal AF1 domain and direct binding of 6-MP with NR4A was not determined. A subsequent study showed that 6-MP induced HIF1α and NR4A1; induction of the former protein was NR4A1-dependent in Hela cells, and this was associated with enhanced new vessel formation in HUVEC cells (Yoo et al., 2007). Other studies also demonstrate that 6-MP activates NR4A1- dependent responses (Qin et al., 2014; Kurakula et al., 2019; Pulakazhi et al., 2021). In addition, it was shown that inhibition of mouse intestinal fibrosis was inhibited by both 6- MP and CsnB, and results from NR4A1^−/−^ and wild-type NR4A1^+/+^ mice indicate that the effects of both compounds were NR4A1-dependent (Pulakazhi et al., 2021). Since the known NR4A1 ligand CsnB binds directly to the receptor then 6-MP may also bind NR4A or somehow mimic the effects of an NR4A1 ligand, and this needs to be determined.

### Cytosporone B (CsnB) and related compounds (Fig. 3B-E)

Wu and coworkers first described the isolation and characteristics of Cytosporone B (CsnB) which bound NR4A1 and stimulated NR4A1-dependent trans-activation and other responses (Zhan et al., 2008). For example, CsnB induced blood glucose levels and liver gluconeogenesis enzymes in wild-type but not NR4A^−/−^ mice. CsnB also inhibited cancer cell growth, and induced apoptosis and the latter response was also due, in part, to nuclear export of NR4A1. Subsequent studies showed that various CsnB analogs were also active as NR4A1 ligands and apoptosis was also induced by transcriptional activation of brain and reproductive organ-expressed protein (BRE), a death receptor associated protein (Liu et al., 2010). Structure-activity studies demonstrate the importance of the ester group and other modifications (Liu et al., 2010; Xia et al., 2013; Yang et al., 2020) and CsnB has been used in several functional studies; for example, as indicated above NR4A1 inhibits TGFβ-induced fibrosis, and this is enhanced by CsnB (Palumbo-Zerr et al., 2015). In contrast, renal interstitial fibrosis in mice with unilateral ureteral obstruction results in the induction of NR4A1 expression and fibrosis and treatment with CsnB further enhances interstitial fibrosis (Tao et al., 2023). Thus, in these different models of fibrosis, the effects of both NR4A1 and CsnB are tissue-dependent.

Wu and coworkers have also synthesized and characterized other NR4A1 ligands based on the CsnB structure, and these include ethyl-2-[2,3,4-trimethoxy-6-(1- octanoyl)]phenyl acetate (TMPA) (Zhan et al., 2012), n-pentyl-2-[3,5-dihydroxy-2- (nonanoyl)]phenyl acetate (PDNPA) (Li et al., 2015), and 1-(3,4,5- trihy-droxyphenyl)nonan-1-one (THPN) (Wang et al., 2014b). The three CsnB analogs were investigated in separate studies and differences in their respective activities were compared in only some assays. TMPA exhibited responses that were the opposite to those observed for CsnB. For example, TMPA-NR4A1 interactions resulted in dissociation of LKB1 from the receptor and nuclear export of LKB from the nucleus resulting in decreased blood glucose levels in diabetic mice whereas CsnB increased blood glucose levels in fasting C57 mice (Zhan et al., 2008). Since this study did not directly compare the mechanisms of action of CsnB and TMPA in the same animal models the mechanistic differences and similarities with respect to their effects on blood glucose levels is unknown and their individual roles as agonists or inverse agonists could not be determined. Interestingly, the CsnB analog PDNPA bound not only NR4A1 but also NR4A2 and NR4A3 however the former two receptors were not further activated by PDNPA. THPN induces nuclear export and mitochondrial dependent autophagic cell death in melanoma cells (Li et al., 2015). In contrast, PDNPA inhibits interaction of NR4A1 with p38α and phosphorylation of the receptor and this results in suppression of NFKβ- dependent transactivation in RAW264.7 cells and lipopolysaccharide-induced inflammation in mouse models (Li et al., 2015).

PDNPA and TMPA interact with similar NR4A1 binding sites however the key amino acids associated with the subsequent interactions of the ligand-bound receptor with p38 or LKB1 were different, and this may have contributed to the observed functional differences between the compounds. In a series of amino acid mutation studies, Wu and coworkers reported the complexities involved in ligand-NR4A1 binding and their interactions with p38 and LKB1 (Zhan et al., 2012; Li et al., 2015; Tao et al., 2023). The T595E mutation in NR4A1 that affected binding to LKB1 (Tao et al., 2023) did not affect binding to p38. For example, both PDNPA and TMPA bind in the same region of NR4A but have distinct functions, however, mutational analysis of amino acids in the LBD of NR4A1 reveal differences in PDNPA-NR4A1 and TMPA-NR4A1 interactions (Li et al., 2015). LBD mutation (C566R and T595E) did not affect p38 nor PDNPA binding but decreased TMPA and LKB1 binding. Mutations of H516W or P597W also differentially affected these interactions and other mutations bound PDNPA but not TMPA (Li et al., 2015). It was concluded that although both TMPA and PNDPA bind a similar region of NR4A1, other factors dictated the different *in vitro* and *in vivo* outcomes of treatment with these two NR4A1 ligands. Treatment of hepatic L02 cells with TMPA induced phosphorylation of AMPK whereas PDNPA had no effect. In contrast PDNPA, but not TMPA suppressed induction of TNFα by LPS. These observations demonstrate that both compounds are SNR4AMs and indicate that the binding and modeling studies of these biological active CsnB analogs does not predict their specific NR4A-dependent biological/functional effects which must be determined in functional assays.

### Fatty acids and prostaglandins (Fig. 4)

**Fig. 4.**
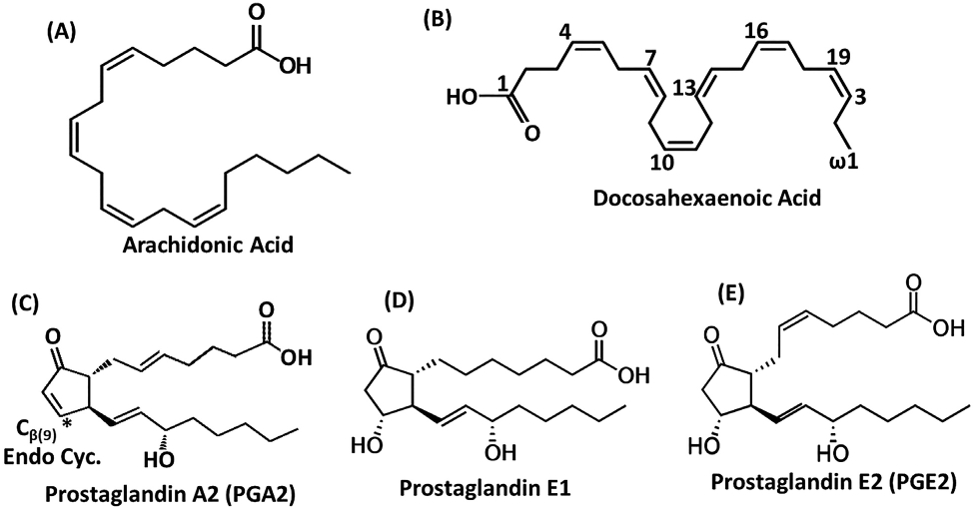
Structures of fatty acid derived NR4A ligands arachidonic acid **(A)**, docosahexaenoic acid **(B)**, prostaglandin A2 **(C)**, prostaglandin E1 **(D)** and prostaglandin E2 **(E)**.

Fatty acids are known ligands for retinoid X receptor, PPARs, FXR, LXR, and NR2E1 (Schroeder et al., 2008; Bedi et al., 2017; Jaladanki et al., 2021; Kandel et al., 2022) and using multiple binding assays, it was also shown that unsaturated fatty acids such as arachidonic acid and docosahexaenoic acid (DHA) (Fig. 4A,B) but not saturated fatty acids, bound NR4A1(LBD) (Vinayavekhin and Saghatelian, 2011). Using NMR spectroscopy, it was shown that DHA bound to the canonical ligand binding pocket of NR4A2 and results also suggest interactions with PIASɣ, a SUMO-E3-ligase that also binds NR4A2. Although DHA decreased NR4A2-dependent transactivation in HEK293T and MN9D cells, the effects were minimal, and the overall functional significance of unsaturated fatty acid bound NR4A2 requires further investigation (de Vera et al., 2016). Prostaglandin A2 (PGA2) (Fig. 4C) was initially identified as a ligand for NR4A3 which induced NR4A3-dependent transactivation (Kagaya et al., 2005) and subsequent studies also showed that PGA2 bound both NR4A2 and NR4A1 (Lakshmi et al., 2019; Rajan et al., 2022). There is also evidence that PGA2 covalently modifies NR4A however the functional and possible endogenous roles for this compound are unclear. The potential endogenous functions of PGE1 and PGE2 (Fig. 4D,E) have recently been reported; both compounds bind NR4A2, inhibit inflammation in neuronal cells and are neuroprotective in the MPTP-induced Parkinson’s Disease mouse model (Rajan et al., 2020). Results of this detailed study suggest the possibility that PGE1/PGA1 may represent bona fide endogenous ligands of Nurr1 (NR4A2) (Rajan et al., 2020).

### Bis-indole derived compounds (CDIMs) (Fig. 5)

**Fig. 5.**
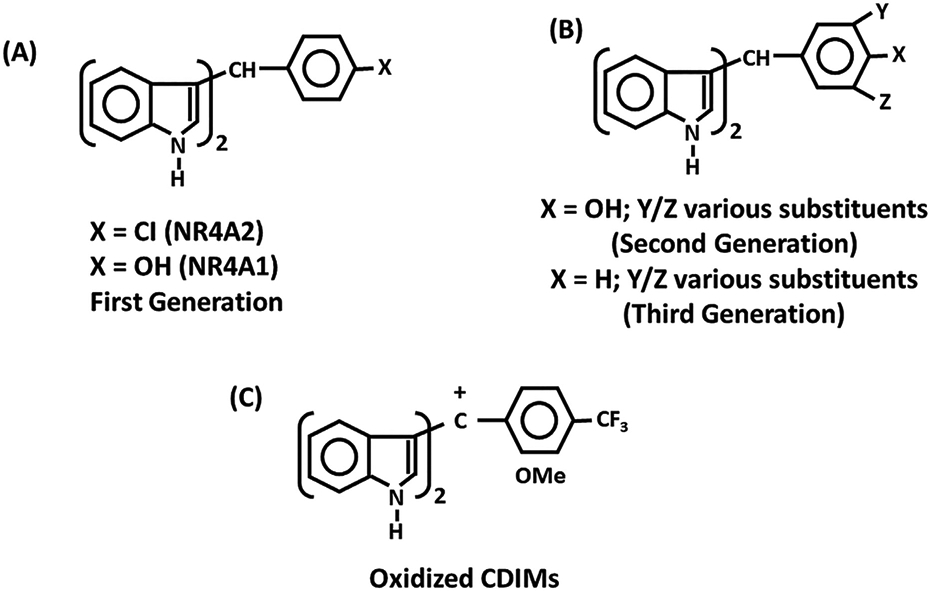
Structures of 4-substituted **(A)** and 3,5-disubstitutedphenyl bis-indole analogs **(B)** that bind NR4A1 or NR4A2; the oxidized analogs **(C)** also bind NR4A1 and exhibit potent anticancer activity (Chen et al., 2019a).

**Fig. 6.**
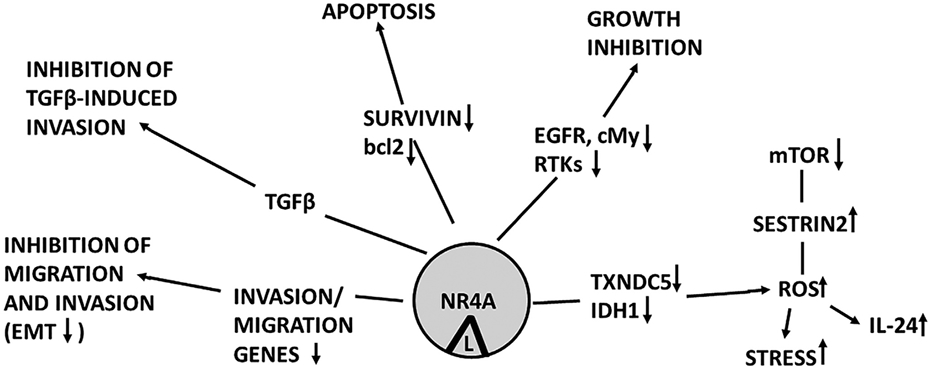
Pathway activated in many solid tumor-derived cells treated with NR4A1 and NR4A2 inverse agonists which block NR4A1/NR4A2-regulated pro-oncogenic genes and pathways (Safe and Karki, 2021).

1,1,-Bis-(3’-indolyl)methane (DIM) is a metabolite of indole-3-carbinol a major chemoprotective agent produced in cruciferous vegetables (Safe et al., 2008). DIM was modified synthetically by adding an additional aromatic group to the methane carbon to form methylene substituted DIMs (CDIMs) (Fig. 5) which were identified as potent inhibitors of mammary tumor growth in a carcinogen-induced rat model (Huggins model) of breast cancer (Qin et al., 2004). Initial studies on a series of 1,1-Bis(3'-indolyl)-1-(p-substitutedphenyl)methane analogs identify some of the compounds as PPARƔ ligands (Qin et al., 2004). Subsequent studies showed that 4-hydroxyphenyl (DIM8) and 4- carbomethoxy-phenyl (DIM14) analogs activated NR4A1-dependent transactivation in cancer cells and also bound NR4A1 in a direct binding assay that measured fluorescent quenching of a Trp in the LBD (Chintharlapalli et al., 2005; Lee et al., 2010, 2014). Both DIM8 and DIM14 were used as NR4A1 ligands in multiple solid tumor derived cancer cell lines and these compounds acted as inverse NR4A1 agonists and inhibited NR4A1-regulated pro-oncogenic genes and pathways as illustrated in Figure 6. These CDIM analogs inhibited cancer cell migration, survival, migration, and invasion in all cancer cell lines investigated however, there was some cell context-dependent variability in the genes that were affected (Lee et al., 2010, 2012, 2014; Hedrick et al., 2015a,b; Hedrick and Safe, 2017; Lacey et al., 2017; Mohankumar et al., 2019) (Fig. 6). For example, the PAX3-FOX01 fusion gene is oncogenic and a major driver of alveolar rhabdomyosarcoma (ARMS) tumor growth in children and in mouse models and DIM8 inhibits NR4A1-regulated PAX3-FOX01 and downstream genes in ARMS cells and in animal models (Lacey et al., 2017). PAX3-FOX01 is primarily expressed in ARMS but not other cancer cell lines. In addition, it was also observed that many of the NR4A1 regulated genes such as survivin, PAX3-FOX01, several integrins and G9A were dependent on formation of NR4A1/Sp1 or NR4A1/Sp4 complexes bound to GC-rich sequences of target gene promoters (Lee et al., 2010; Hedrick and Safe, 2017; Lacey et al., 2017; Karki et al., 2020b; Shrestha et al., 2021a). Thus, NR4A1 acts as a cofactor for several pro-oncogenic Sp-regulated genes and DIM8 and other inverse NR4A1 agonists inhibit or block the cofactor functions of NR4A1 and decrease expression of selected Sp-regulated genes. This mechanism is commonly observed for gene regulation by several NRs (Safe and Kim, 2008).

*In vivo* studies showed that IC_50_ values for tumor growth inhibition in mouse xenograft models by DIM8 or DIM14 ranged from 20-40 mg/kg/day and pharmacokinetics indicated that in mice treated with DIM8, the serum levels of this compound were low due to rapid metabolism (De Miranda et al., 2013). In order to decrease the metabolism of DIM8 several buttressed analogs containing 3 or 3- and 5-substituents, which were ortho to the 4-hydroxyl group and thereby could sterically inhibit metabolic conjugation of the hydroxyl substituent were synthesized. These “DIM8–3,5” compounds bind NR4A1 and among those compounds that have been tested they are more potent inhibitors of breast tumor growth (IC_50_ ~ 2-10 mg/kg/day) compared to DIM8 (Hedrick et al., 2019). Presumably, the enhanced anticancer activities are due, in part, to longer serum half-lives of DIM8-3,5 analogs however, this has not yet been confirmed. Ongoing studies with a series of 1,1-Bis(3’-indolyl)-1-(dichlorophenyl)methane analogs showed that the hydroxyl group was not necessary for binding NR4A1 and the 3,5-dichlorophenyl analog (DIM-3,5- CI_2_) was an NR4A1 ligand (Karki et al., 2021). Moreover, among a group of 4 DIM-3,5 analogs (DIM-3-Br-5-OCH_3_, DIM-3-CI-5-OCF_3_, DIM3-CI-5-CF_3_ and DIM-3-Br-5-OCF_3_) all of these compounds inhibited breast tumor growth at doses of <1 mg/kg/day and represented a third generation set of CDIM analogs that are being developed for future clinical trials. Oxidized DIM compounds have also been investigated and are potent inhibitors of tumor growth. These compounds are similar to the CDIMs in structure but act to induce nuclear export of NR4A1 which forms a mitochondrial proapoptotic complex with bcl2 (Chen et al., 2019a).

Initial evaluation of the binding of the 4-substitutedphenyl CDIM analogs identified the 4-chlorophenyl analogs (DIM12) as a compound that modulated NR4A2-dependent transactivation (Li et al., 2012, 2019). Modeling studies show that this ligand preferentially bound a cofactor site outside the LBD, and it has been confirmed that DIM12 does not interact with the LBD of NR4A2 nor NR4A1 (Hammond et al., 2018). In solid tumors DIM12 acts as an inverse agonist to inhibit pro-oncogenic NR4A2-dependent tumor growth, survival, migration, and invasion (Karki et al., 2020a) and in neuronal models of inflammation and Parkinson’s disease (PD) DIM12 inhibits inflammation (De Miranda et al., 2015; Hammond et al., 2015, 2018; Hu et al., 2017). It is noteworthy that DIM12, which contains a 4-chlorophenyl moiety, binds the cofactor site of NR4A2 (Hammond et al., 2018) whereas in ongoing studies we observed that a series of dichlorophenyl analogs (including DIM-3,5-CI2) interact with the binding pocket of NR4A1. In contrast, the studies with CDIMs show that among those compounds previously reported none of them interact with NR4A3.

### Celastrol and related compounds

**Fig. 7.**
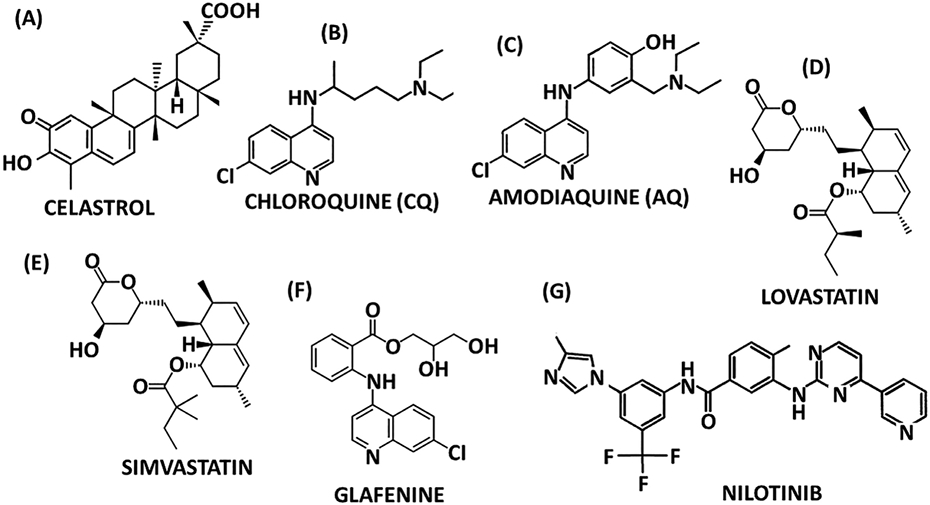
Drugs that exhibit NR4A activity include celastrol (NR4A1) **(A)**, chloroquine (NR4A2) **(B)**, amodiaquine (NR4A2) **(C)**, lovastatin (NR4A2) **(D)**, simvastatin (NR4A2) **(E)**, glafenine (NR4A2) **(F)** and nilotinib (NR4A1) **(G)**.

Celastrol (Fig. 7A) is a pentacyclic triterpenoid and a potent anticancer agent that binds NR4A1 with a K_D_ of 0.29 μM and inhibits NR4A1-dependent transactivation: NR4A1 is involved in celastrol-dependent inflammation and induction of autophagy in mice (Hu et al., 2017). Interestingly NR4A2 also regulates autophagy and DIM12 decreases ATG7 and ATG12 gene expression in pancreatic cancer cell and tumors (Zarei et al., 2021), whereas celastrol induced NR4A1-dependent autophagy, demonstrating opposing effects of NR4A1 and NR4A2 ligands which may also be cell context dependent. However, mechanistically the anti-inflammatory effects observed were associated with celastrol-induced nuclear export of NR4A1 and subsequent interaction of NR4A1 with tumor necrosis factor receptor associated factor 2 (TRAF2) which inhibits inflammation by promoting mitochondrial ubiquitination and autophagy (Hu et al., 2017). Subsequent structure-activity studies with a series of synthetic celastrol analogs identified an A-ring aromatized derivative with a K_D_ = 0.87 μM as a possible new lead compound (Chen et al., 2019b). Celastrol is only one of many naturally occurring triterpenoid anticancer agents and it is likely that some of these compounds may also be NR4A ligands.

### Drugs and related compounds

A cell-based assay was used to screen a chemical library of 960 FDA drugs and two antimalarial drugs, amodiaquine and chloroquine and a pain-relieving drug, glafenine, were identified as activators of NR4A2-dependent transactivation. All of these compounds shared a common scaffold, namely 4-amino-7-chloroquinoline. Amodiaquine and chloroquine were specific NR4A2 ligands that inhibited induced inflammation in neuronal cells, enhanced NR4A2-dependent dopaminergic effects in rat cell models (Kim et al., 2015) and also in an *in vivo* model of Parkinson’s Disease (PD) (Kim et al., 2015) where rats are treated with 6-hydroxydopamine. In addition, amodiaquine also improved behavioral deficits in this same model of PD and enhanced cognition in adult C57BL/6 mice (Kim et al., 2016). A second study showed that amodiaquine inhibited DSS-induced colitis and this was accompanied by NR4A-dependent induction of CD25^+^:Foxp3^+^ regulatory T cell development (Won et al., 2017). Subsequent *in vitro* studies in 293T cells report that amodiaquine activated NR4A1- NR4A2- and NR4A3-dependent transactivation suggesting that this compound may be a unique pan-NR4A ligand. There is also evidence that the NR4A2-dependent effects of amodiaquine and chloroquine are variable and cell-context dependent (Won et al., 2017) and like many NR4A ligands, they are selective receptor modulators (Munoz-Tello et al., 2020). 4-Amino-7-chloroquinoline has also been used as a scaffold for synthesizing other NR4A2 ligands and one of the most active compounds was 4-amino-8-chloro-2-methylquinoline which contained a free amino group (Willems et al., 2021).

A recent study showed that several commercially available statins including Fluvastatin, lovastatin, simvastatin, atorvastatin, rosuvastatin and pitavastatin activated NR4A2-dependent transactivation and reduced inflammatory response in neuronal- derived cells (Willems et al., 2022). Results of analysis of gene expression data from female non-smoking lung cancer patients identified NR4A1 as a potential target gene and they demonstrated that nilotinib, a protein-tyrosine kinase inhibitor, was also an NR4A1 ligand that inhibited lung cancer cell growth (Sun et al., 2019). Nilotinib is a polycyclic compound containing multiple heterocyclic rings and could be another potential scaffold for developing new NR4A ligands.

### Flavonoids and other natural/microbial products (Fig. 8)

Flavonoids are highly expressed in fruits, nuts, and vegetables and their consumption correlates with multiple improved health outcomes and these compounds have also been used for chemotherapy (Safe et al., 2021). The anticancer activities of flavonoids have been extensively reported and in one study the flavonoid kaempferol downregulated expression of G9a in gastric cancer cells (Kim et al., 2018). Previous studies show that G9a is an NR4A1-regulated gene in Rhabdo-myosarcoma cells (Shrestha et al., 2021a) suggesting that kaempferol and other flavonoids may also bind NR4A1. Initial studies showed that both quercetin and kaempferol bound NR4A1 and *in vitro* studies confirmed that both compounds exhibited inverse NR4A1 agonist activities in rhabdomyosarcoma cells and inhibited tumor growth in an athymic mouse xenograft model (Zhang et al., 2023). Anti-inflammatory results were also observed for these flavonoids in endometriotic cells (Shrestha et al., 2021b). A more extensive study of flavone and 19 hydroxyflavones showed that they all bound NR4A1, however there was not an obvious structure-binding or structure-activity relationship with respect to the substitution pattern or number of hydroxyl groups in the chromone or phenyl rings (Lee et al., 2023). Using a direct binding loss of fluorescence assay K_D_ values for binding NR4A1 ranged from 0.36 μM for 3,5,7-trihydroxyflavone (galangin) to 45.8 μM for 3’-hydroxyflavone. In contrast, KD values were much lower using ITC and a KD of 0.001 μM was determined for 3,5,7-trihydroxyflavone. Structure-binding and structure-activity relationships among that hydroxyflavones were not evident and it was concluded that these compounds were selective NR4A1 modulators. A series of adamantly-flavonoid derivatives also bind NR4A1 and are effective as anti-inflammatory agents. One of these compounds (B7) bound NR4A1 with a K_D_ value=0.355 μM and induced mitochondrial localization of the receptor (Ao et al., 2022). A second adamantly analog (B6) that bound NR4A1 inhibited ER stress in bronchial epithelial cells and induced localization of the receptor to the endoplasmic reticulum (ER) (Chang et al., 2023). The flavonoid-derived (chalcone) natural product broussochalcone induced apoptosis/ER stress in pancreatic cancer cells (Lee et al., 2021) however the NR4A1 nuclear or extranuclear mode of action of this compound was not reported.

A search for possible dopamine metabolites that may interact with NR4A2 in the brain identified 5,6-dihydroxyindole (DHI), a dopamine metabolite as an NR4A2 ligand that covalently crosslinks with the receptor (Bruning et al., 2019). Subsequent studies show that several isomeric chloro- and bromoindole isomers and 5,6-dihaloindoles also bound NR4A2 with K_D_ values in the low (1-3) μM ranges (Kholodar et al., 2021). Although functional responses have been observed for DHI in cell culture and zebrafish models a role for DHI in neuronal homeostasis and under disease conditions has not been determined. A number of other structurally-diverse natural products have been characterized as compounds that bind and/or activate NR4A1 or NR4A2. Several compounds including the bile acid metabolite isoallolithocholic acid (Li et al., 2021a,b), polyunsaturated fatty acid alkanolamine derivatives (Fang et al., 2020), isoalantoactone (Jung et al., 2019), the alkaloid tetrandine (Lee et al., 2022), resveratrol (Zhang et al., 2022), and 12-deacetyl-12-epi-scalaradial, a marine sesterterpenoid (Zhou et al., 2020) interact with and/or regulate NR4A1-dependent genes and activities. GPA peptide, a fish skin gelatin hydrolysate (Gly-Pro-Ala) is also an NR4A1 ligand (K_D_=7.53 μM) (Deng et al., 2020). GPA inhibits NFKβ-mediated inflammation and attenuates DSS-induced colonic inflammation in a mouse model due, in part, to activation and increased expression of NR4A1. These results obtained with structurally variable natural products demonstrate that NR4A1 and NR4A2 are highly promiscuous nuclear receptors in terms of their ligands and provide different scaffolds that can be used for producing more potent ligands for therapeutic applications.

### Drug screening for NR4A ligands

Several studies have also used chemical library screening assays to identify NR4A ligands. Zhang and coworkers investigated more than 200,000 small molecules from the Specs compound library using a protein structure-guided virtual screening approach and identified 2-imino-6-methoxy-2H-chromene-3-carbo-throamide (IMCA) as a potential NR4A1 target. IMCA exhibited inverse NR4A1 agonist activity that inhibited growth and survival of medullary thyroid cancer cells. However, NR4A1 binding and effects on NR4A1-dependent transactivation were not determined, and it was pointed out that IMCA may act, in part, by inducing nuclear export of the receptor (Zhang et al., 2018). Sitemap suite in Schrodinger software was used to identify small molecule interactions with the LBD of NR4A1 and this resulted in identification and synthesis of several quinoline derivatives (Li et al., 2020, 2021a,b). In the first study, the lead candidate (E)-5-[(8-methoxy-2-methylquinolin-4-yl)amino]-N’-(4-methylthio)benzylidene-1H-indole- carbohydrazide bound NR4A1 with a KD of 3.58 μM and was highly cytotoxic to cancer cell lines (Li et al., 2020). Several other active quinoline derivatives were also NR4A1 ligands with low μM KD values (Li et al., 2021a,b) and results of both studies show that the quinoline derivatives induce apoptosis through nuclear export of NR4A1 to the mitochondria (Li et al., 2020, 2021a,b). Another scaffold for NR4A1 ligands was discovered using the ChemDiv database and virtual screening, and the lead molecule (compound 13) bound NR4A1 with a K_D_ value of 4.03 μM and a synthetic analog exhibited an even lower K_D_ of 0.54 μM (Ding et al., 2021). Modeling/SAR studies were used to identify the NR4A2 ligand isoxazolo-pyridinone (Hintermann et al., 2007) which was active *in vivo* as an inhibitor of experimental autoimmune encephalomyelitis in mice (Montarolo et al., 2014) and Dubois and coworkers (2006) found that several benzimidazoles were potent NR4A2 ligands.

Prostaglandin A2 was initially identified as an NR4A3 ligand (Maruyama et al., 1998) and recent studies on identification of ligands for this receptor probed a drug fragment library using a GAL4 hydrid system (Zaienne et al., 2022). This assay identified methyl indole-3-carboxylate, substituted phenylpiperazines and 3-(4-chlorophenyl) propionic acid as NR4A3-active compounds and these structures can also be used as scaffolds to optimize their activity as ligands for NR4A3.

## Summary and conclusion

NR4A1, NR4A2 and NR4A3 bind structurally diverse compounds that activate or inactivate downstream genes and pathways. The lack of precise structure-binding relationships for these orphan receptors is similar to that observed for some non-orphan receptors such as estrogen receptor-α (ERα, ESR1) which also bind chemicals with diverse structures. A major difference between NR4A and ERα is the lack of an endogenous ligand with binding affinities in the low μM range for the former receptors. Research on Cytosporone B and related compounds demonstrates that these NR4A1 ligands exhibit tissue-specific agonist or inverse agonist activities suggesting that these ligands and others are selective receptor modulators, and this has also been observed for ERα and other NRs. A few reports show that some compounds such as PGA2 and PDNPA bind multiple receptors however their activity tends to be receptor-specific, and this may also be tissue-specific. The identification of more potent NR4A ligands targeting one or more NR4A members remains a challenge and an opportunity for developing more effective agents for treating inflammatory and other diseases where NR4A is a potential drug target.

## Figures and Tables

**Fig. 8.**
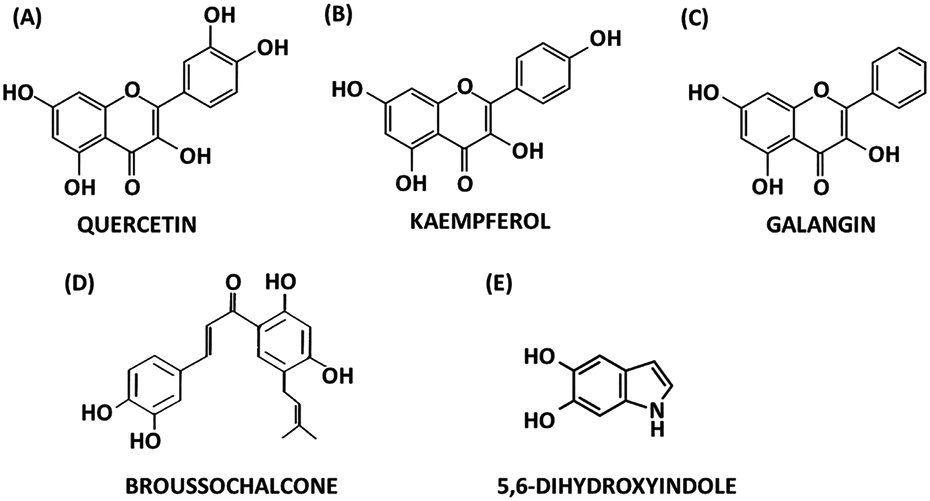
Structures of natural products that bind NR4A include quercetin (NR4A1) **(A)**, kaempferol (NR4A1) **(B)**, galangin (NR4A1) **(C)**, broussochalione (NR4A1) **(D)** and 5,6-dihydroxyindole (NR4A2) **(E)**.
